# Association of *MDM2* SNP309 and *TP53* Arg72Pro polymorphisms with risk of endometrial cancer

**DOI:** 10.3892/or.2013.2433

**Published:** 2013-04-29

**Authors:** TOMOKO YONEDA, AYUMI KUBOYAMA, KIYOKO KATO, TATSUHIRO OHGAMI, KANAKO OKAMOTO, TOSHIAKI SAITO, NORIO WAKE

**Affiliations:** 1 Department of Obstetrics and Gynecology, School of Medicine, Kyushu University, Fukuoka 812-8582, Japan; 2 Gynecology Service, National Kyushu Cancer Center, Fukuoka 811-1395, Japan

**Keywords:** *MDM2* SNP309, *TP53* Arg72Pro, *ESR1 Pvu*II *Xba*I, *p21* codon 31, endometrial cancer

## Abstract

The incidence of endometrial cancer, a common gynecological malignancy, is increasing in Japan. We have previously shown that the ER/MDM2/p53/p21 pathway plays an important role in endometrial carcinogenesis. In the present study, we investigated the effects of germline single nucleotide polymorphisms in murine double minute 2 (*MDM2)* SNP309, *TP53* Arg72Pro, *ESR1 Pvu*II and *Xba*I, and *p21* codon 31 on endometrial cancer risk. We evaluated these polymorphisms in DNA samples from 125 endometrial cancer cases and 200 controls using polymerase chain reaction-based restriction fragment length polymorphism. The association of each genetic polymorphism with endometrial cancer was examined by the odds ratio and 95% confidence interval, which were obtained using logistic regression analysis. The SNP309 GG genotype non-significantly increased the risk of endometrial cancer. The 95% confidence interval for the GG genotype vs. the TT genotype of *MDM2* SNP309 was 1.76 (0.93–3.30). Endometrial cancer was not associated with tested SNP genotypes for *TP53*, *ESR1* and *p21*. The combination of SNP309 GG + TG and *TP53* codon 72 Arg/Arg significantly increased endometrial cancer risk. The adjusted OR was 2.53 (95% confidence interval, 1.03–6.21) and P for the interaction was 0.04. This result was supported by *in vitro* data showing that endometrial cancer cell lines with the SNP309 G allele failed to show growth inhibition by treatment with RITA, which reduces p53-MDM2 binding. The presence of the SNP309 G allele and *TP53* codon 72 Arg/Arg genotype is associated with an increased risk of endometrial cancer in Japanese women.

## Introduction

The incidence of endometrial cancer is increasing; it is the second most common gynecologic malignancy in Japan. Increased estrogen exposure, particularly unopposed estrogen, is a major risk factor for endometrial cancer. Early menarche, infertility, obesity and late menopause are also associated with increased risk of the cancer. Endometrial cancer is broadly classified into one of two clinicopathological types: I and II (1). The former is estrogen-related and occurs in both premenopausal and postmenopausal women. Histologically, it is of the endometrioid type, generally of low cellular grade and has a favorable prognosis. It is frequently preceded by endometrial hyperplasia. These tumor cells frequently express the estrogen receptor (ER), particularly ERα. Type I endometrial cancer development is associated with a variety of genetic alterations, including *PTEN* inactivation, K-ras mutation, β-catenin mutation and microsatellite instability. Type II endometrial cancer is non-estrogen-related and occurs primarily in postmenopausal women. Type II encompasses non-endometrioid histologies and is represented by serous or clear-cell adenocarcinoma. It is commonly associated with an atrophic rather than a hyperplastic endometrium. The cells are negative for ER and the progesterone receptor (PR); type II endometrial cancer exhibits a high cellular grade and is associated with poor prognosis. Genetic alterations in type II carcinomas include p53 mutation, p16 inactivation, HER-2/neu overexpression and reduced E-cadherin expression (1,2.)

A human homologue of the murine double minute 2 (*MDM2*) gene (also known as HDM2 in humans) is frequently overexpressed in several types of human cancer, particularly in breast carcinomas and soft tissue sarcomas (3). The major contribution of MDM2 to cancer development is through tight inhibition of tumor suppressor p53 activity and stability. Biochemically, MDM2 functions as an E3 ubiquitin ligase responsible for p53 ubiquitination and degradation (4–6). Recent studies have identified numerous additional MDM2-interacting proteins and p53-independent functions of MDM2 in the regulation of multiple signaling pathways, including the pRb/E2F complex and the PI3K/Akt pathway (7). MDM2 overexpression is also strongly related to the presence of ER (8,9.) Elevated ER expression in cells lacking ER induces MDM2 overexpression (3). We have shown that overexpression of wild-type ERα in NIH3T3 cells results in a significant increase in MDM2 protein levels. *MDM2* gene expression is also regulated by the Ras-driven Raf/MEK/MAP kinase pathway in a p53-independent manner (10). We have also demonstrated that the Ras/ER/MDM2 pathway is critical for NIH3T3 cell transformation (11,12) and that blockage of this pathway by inhibitors or *MDM2* siRNA induces p53 and p21 expression and suppresses cell proliferation in estrogen-dependent cancer such as endometrial and ovarian cancer (13). These results suggest that the ER/MDM2/p53/p21 pathway plays an important role in the development of endometrial cancer.

A single nucleotide polymorphism (SNP309 T>G; rs2279744) has been identified in the *MDM2* promoter. The SNP309 G allele has high affinity for the transcriptional activator SP1, which results in a higher level of *MDM2* mRNA and MDM2 protein and subsequent attenuation of the p53 pathway. In humans, the SNP309 G allele is associated with accelerated tumor formation in hereditary cancer associated with Li-Fraumeni syndrome and sporadic soft tissue sarcomas (14). SP1 is a well-characterized co-transcriptional activator of multiple hormone receptors, including ER. In an analysis of 3 different types of sporadic cancer (diffuse large B-cell lymphoma, soft-tissue sarcoma and invasive ductal breast carcinoma), Bond *et al*(15) showed that the SNP309 G allele is associated with gender-specific and hormone-dependent acceleration of tumorigenesis. Several studies, however, found no evidence of an association between SNP309 and cancer risk (16–18). The results remain controversial.

The p53 signaling pathway is activated by a wide variety of stress signals. Activation of p53 induces growth arrest, cellular senescence and apoptosis (19). Numerous polymorphisms are present in the *TP53* locus. A well-known G to C SNP at *TP53* codon 72 results in an arginine (Arg; CGC) or proline (Pro; CCC) polymorphism (rs1042522). This polymorphism is of particular interest owing to its functionality, although its biological function is controversial.

*ESR1* is the principal ER expressed in the endometrium and is thought to be important in the development of endometrial carcinoma. The *ESR1* gene contains several SNPs whose functional significance remains unknown. The two most frequently studied polymorphisms located in *ESR1* gene intron 1 are often identified by their restriction endonucleases, *Pvu*II T/C (rs2234693) and *Xba*I A/G (rs9340799).

p21, a cyclin-dependent kinase inhibitor, is the major downstream component of the TP53 tumor suppressor pathway. This protein binds to and inhibits all cyclin-dependent kinase complexes, causing cell cycle arrest in G1. The p21 codon 31 C/A (rs1801270) in exon 2 leads to a serine (Ser)/Arg amino acid substitution and is located in the DNA-binding zinc-finger motif of the gene (20). This polymorphism has been implicated in cervical adenocarcinoma (21) and endometrial cancer (22,23).

The *MDM2* SNP309 G/G genotype increases the risk of endometrial cancer in Caucasians (24,25) and in Japanese women (26). Furthermore, a combination of the homozygous Arg/Arg genotype of *TP53* codon 72 and the homozygous GG genotype of *MDM2* SNP309 is significantly associated with the risk of endometrial cancer in Japanese women.

We performed the present case control study to investigate the relationship between endometrial cancer risk and multiple genetic polymorphisms, including *MDM2* SNP309, *TP53* Arg72Pro, *ESR1 Pvu*II and *Xba*I, and *p21* codon 31, in Japanese women.

## Materials and methods

### Study subjects

Subjects included 125 endometrial cancer patients who were diagnosed at the Department of Obstetrics and Gynecology of Kyushu University Hospital and Kyushu Cancer Center Hospital between 1993 and 2010. All patients provided informed consent. There was no family history of endometrial cancer in any of these cases. Control subjects were selected from 7,132 women aged 49–76 years, living in the East Ward of Fukuoka City, who had completed a baseline survey between February 2004 and August 2007 of an on-going cohort study regarding lifestyle-related diseases. Out of 2,055 women aged 49–60 years, 2,783 women aged 60–69 years, and 1,147 women aged ≥70 years who had no history of cancer and who had donated a blood sample for a genetic study under signed informed consent, 106, 66 and 28 women, respectively, were randomly selected in proportion to the age distribution of the 125 cases of endometrial cancer accrued as of the end of March 2010.

### Genotyping

Genomic DNA was extracted from 10-ml blood samples using the QIAamp^®^ DNA Blood Maxi kit (Qiagen, Hilden, Germany). DNA of the controls was extracted using an automated DNA isolation system (NA-300; Kurabo, Osaka, Japan). We performed RFLP using the digested PCR products, which were electrophoresed in agarose gels and visualized using ethidium bromide. *MDM2* genotyping was performed as previously described (24) using the primers 5′-CGGGAGTTCAGGGTAAAGGT-3′ (sense) and 5′-AGCAAGTCGGTGCTTACCTG-3′ (antisense). The PCR product of 352 bp was digested with MspA1 (New England Biolabs, Ipswich, MA, USA). A *TP53* codon 72 genotyping assay was performed as previously described (27) using primers 5′-TTGCCGTCCCAAGCAATGGATGA-3′ (sense) and 5′-TCTGGGAAGGGACAGAAGATGA-3′ (antisense). The restriction enzyme *Bstu*1 (New England Biolabs) was used to digest the 199-bp PCR product. The *ESR1* gene polymorphisms were investigated using the primers 5′-CTGCCACCCTATCTGTATCTTTTCCTATTC TCC-3′ (sense) and 5′-TCTTTCTCTGCCACCCTGGCGTC GATTATCTGA-3′ (antisense). The 1374-bp PCR product was digested using the restriction endonuclease *Xba*I (Takara; Shiga, Japan), or *Pvu*II (Takara) (28,29). Genotyping for the *p21* polymorphism was performed according to a previously published method (30,31). A 245-bp fragment was amplified using the primer sets 5′-ATAGTGTCTAATCTCCGCCG-3′ (sense) and 5′-AAGTCACCCTCCAGTGGTGT-3′ (antisense). The 245-bp PCR product was digested using *Blp* 1 (New England Biolabs). To test the reliability of these assays, genotypes of the *MDM2*, *TP53*, *ESR1* and *p21* polymorphisms were validated using direct sequencing for 30 randomly selected samples of cases and controls (10 of each different genotype). The SNP309 sequencing primers used to amplify a 405-bp sequence were 5′-GATTTCGGACGGCTCTCGCGGC-3′ (sense) and 5′-AGCAAGTCGGTGCTTACCTG-3′ (antisense). The *ESR1 Pvu*II and *Xba*I sequencing primers used to amplify a 584-bp sequence were 5′-AGGTTTATGCAATGACG-3′ (sense) and 5′-TCCTTGGCAGATTCCATGGC-3′ (antisense). Sequencing reactions were conducted using BigDye Terminator version 3.1 (Applied Biosystems, Foster City, CA, USA).

### Cell culture

Endometrial cancer cell lines were maintained in DMEM supplemented with 10% FBS for HHUA and 15% FBS for Hec6 and Sawano cells.

### Cell growth assay

Cells were plated in a 6-cm dish at 1×10^5^ cells/dish and incubated with a medium supplemented for 24 h. Subsequently, to reactivate p53 and induce tumor cell apoptosis, the compound RITA (Tocris, Ellisville, MO, USA), was added, and the cells were further incubated for 48 and 96 h. Following incubation, floating cells were washed away and adherent cells were detached from the dishes using 0.25% trypsin. Detached cells were then counted using a hemocytometer.

### Statistical analysis

Comparisons of means and proportions between cases and controls were performed using the t-test and Chi-square test, respectively. Deviation from the Hardy-Weinberg equilibrium was evaluated using the Chi-square test with 1 degree of freedom. The association of each genetic polymorphism with endometrial cancer was examined by the odds ratio (OR) and the 95% confidence interval (95% CI), which were obtained using logistic regression analysis. The 95% CI was derived from the standard error of the logistic regression coefficient. Statistical adjustment was made for age and body mass index (BMI) with continuous variables for each included as covariates. Trends in ORs according to the number of minor alleles were evaluated using the Wald test. For analyzing the interaction between two polymorphisms, heterozygous and homozygous genotypes of the minor alleles were combined and statistical evaluation was conducted using the Wald test for the product term of the two variables representing the genotypes containing the minor allele. Statistical significance was declared if a two-sided P-value <0.05 or if the 95% CI did not include unity. Statistical analyses were carried out using SAS version 8.2 (SAS Institute Inc., Cary, NC, USA).

## Results

### Characteristics of endometrial cancer cases and controls

Selected characteristics of endometrial cancer cases and controls are shown in Table I. The mean age in the cases group was lower than that of the control group as the latter were selected from women aged 49–76 years, while the former were recruited regardless of age. BMI was significantly greater in cases than in controls. A total of 49 (39.2%) overweight women (BMI ≥25 kg/m^2^) were included in the cases and 38 (19.0%) in the controls (P<0.001). Most tumors (112, 89.6%) were of endometrioid histology. Most cases were of The International Federation of Gynecology and Obstetrics (FIGO) stage I (95, 76.0%).

Frequencies of variant alleles between cases and controls respectively were as follows: SNP309 G allele (0.516 and 0.445), *TP53* codon 72 Pro allele (0.364 and 0.370), *ESR1 Pvu*II C allele (0.456, 0.445), *ESR1 Xba*I G allele (0.216, 0.205), and *p21* codon 31 Ser allele (0.460 and 0.463). Genotype distributions of the SNP309, *TP53* Arg72Pro, *ESR1 Pvu*II and *Xba*I, and *p21* codon 31 polymorphisms in the controls did not deviate from Hardy-Weinberg equilibrium.

### The MDM2 SNP309, TP53, ESR1 and p21 polymorphisms do not individually increase the risk of endometrial cancer development

Table II shows the association between endometrial cancer risk and SNP309, *TP53*, *ESR1* and *p21* polymorphisms. The OR for the SNP309 GG genotype when compared with the SNP309 TT genotype non-significantly increased risk. The crude OR was 1.76 (95% CI, 0.93–3.30), and the age- and BMI-adjusted OR was 1.64 (95% CI, 0.81–3.28). There was also no measurable association between endometrial cancer and the *TP53* Arg72Pro, *ESR1 Pvu*II and *Xba*I, or the *p21* codon 31 polymorphisms. Adjustment for age and BMI did not significantly alter the results. The analysis was repeated with stratification by menopausal status, histological status (Table III) and overweight status. There were 261 postmenopausal women (78 cases and 183 controls) and 102 women with type I endometrial cancer. For SNP309, the OR associated with the G allele, a slight increase was seen with postmenopausal status and type I status in cases that were not statistically significant. When the analysis was confined to overweight women (49 cases and 38 controls), the adjusted SNP309 OR for GG vs. TT was 2.39 (95% CI, 0.59–9.66).

### Combination of the MDM2 SNP309 G allele and the Arg/Arg genotype of TP53 codon 72 interacts significantly to affect the risk of endometrial cancer

In the analysis of the combination of the two polymorphisms (Table IV), the SNP309 TG and GG genotypes as well as the *TP53* Arg/Pro and *TP53* Pro/Pro genotypes were combined. A significant increase in the adjusted OR associated with the SNP309 G allele was limited to those homozygous for the *TP53* Arg allele (OR, 2.53; 95% CI, 1.03–6.21). A statistically significant interaction was observed between the 2 polymorphisms on the risk of endometrial cancer (P for the interaction=0.04). We repeated the analysis to include postmenopausal status and type I endometrial cancer status. Adjusted ORs for postmenopausal women and type I women possessing both polymorphisms when compared with being homozygous for both wild-type alleles were 2.96 (95% CI, 1.04–8.44) and 2.51 (95% CI, 0.97–6.53), respectively. There was a statistically significant interaction between the two polymorphisms regarding the risk of endometrial cancer (P for the interaction=0.03 and 0.01, respectively). Moreover, the corresponding value for type I among postmenopausal women (59 cases) was 3.24 (95% CI, 1.03–10.16), which reflected a statistically significant interaction between the SNP309 and *TP53* Arg72Pro polymorphisms (P for the interaction=0.009).

### Combination of the TP53 72Pro allele and homozygosity for the p21 codon 31 Ser allele is associated with a decreased risk of endometrial cancer

We further examined the effect of SNP309 combined with *ESR1* or *p21* and SNP309 with both *TP53* and *p21* polymorphisms on the risk of endometrial cancer (Table V). No significant differences were observed between the combination of the SNP309 and the *Pvu*II or *Xba*I or *p21* polymorphism. The combination of having the *TP53* 72Pro allele and homozygosity for the *p21* codon 31 Ser allele, however, was associated with a decreased risk of endometrial cancer (crude OR, 0.28; 95% CI, 0.09–0.92). A statistically significant interaction was observed between the 2 polymorphisms for a decreased risk of endometrial cancer (P for the interaction=0.04), although there was no significant difference once adjustment was made for age and BMI.

### The SNP309 GG genotype abrogates the cytostatic effect of RITA on tumor cells

MDM2 overexpression is observed in multiple malignancies. Due to the importance of the p53-MDM2 interaction, restoration of p53 activity by inhibiting MDM2 binding represents a novel antineoplastic strategy. RITA is a low-molecular-weight compound previously identified in a cell-based screen for wild-type p53-reactivating compounds. RITA binds to the amino terminus of p53, inhibiting p53 binding to MDM2 in cultured cells and in human tumor xenografts *in vivo*. This results in derepression of p53 and highly efficient induction of apoptosis (32).

**Figure 1:**
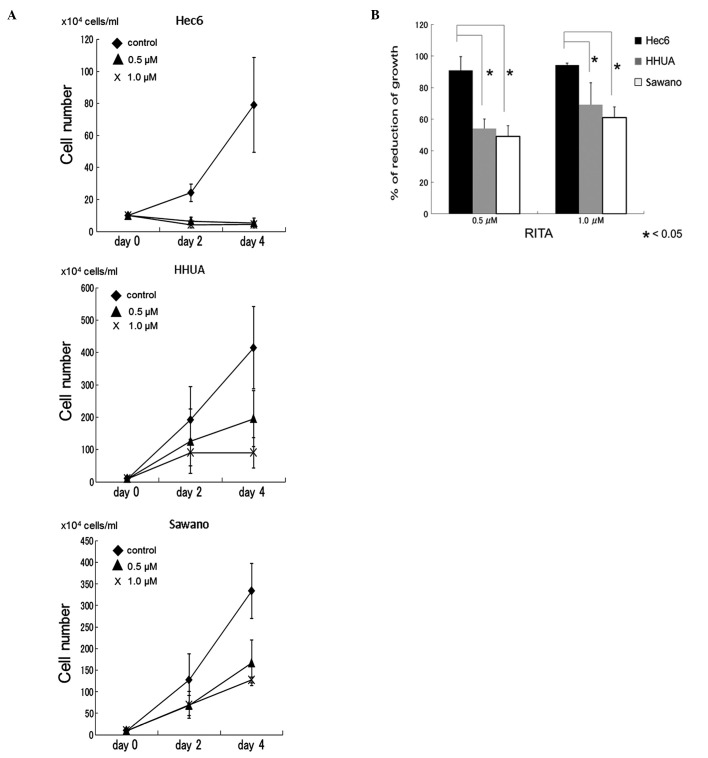
Growth inhibitory effects of RITA on endometrial cancer cell lines. (A) Treatment with RITA inhibited cell growth in endometrial cancer cell lines. Following treatment with DMSO (control) or 0.1 or 0.5 μM RITA for two or four days for three endometrial cancer cell lines (Hec6, HHUA and Sawano), viable cells were counted. Experiments were performed in triplicate. Vertical bars indicate SDs. (B) The SNP309 G allele inhibited the cytostatic property of RITA in tumor cells. Following treatment with DMSO and RITA for four days, viable cells were counted and the reduction in the rate of growth was calculated. Vertical bars indicate SDs. ^*^P<0.05.

We demonstrated that a combination of the SNP309 G allele and the homozygous Arg/Arg genotype of *TP53* codon 72 was associated with an increased risk of endometrial cancer (Table IV). These results suggest that polymorphisms of *MDM2* and *TP53* may influence the effect of RITA. We therefore, assessed the growth-suppressive effect of RITA on three endometrial cancer cell lines, Hec6, HHUA and Sawano, *in vitro*. All three lines express wild-type p53. SNP309 and *TP53* Arg72Pro polymorphisms were analyzed in these lines with PCR-RFLP and direct sequencing. Hec6 cells were SNP309 TT homozygous and *TP53* Arg72 homozygous, HHUA cells were SNP309 TG heterozygous and *TP53* Arg72 homozygous, and Sawano cells were SNP309 GG homozygous and *TP53* Arg72Pro heterozygous (Table VI). Treatment with RITA (0.5 and 1.0 μM) suppressed cell growth in all cell lines in a dose-dependent manner (Fig. 1A). Following treatment with 0.5 μM RITA for four days, the rates of inhibition were: Hec6 cells, 90.9±8.7%; HHUA cells, 54.1±6.0%; and Sawano cells, 49.0±15.8%. Following treatment with 1.0 μM RITA, these rates were 94.3±1.2%, 79.0±4.6% and 61.0±6.7%, respectively. The inhibitory effect was significantly less pronounced in HHUA and Sawano cells with the SNP309 G allele compared to that in Hec 6 cells carrying the SNP309 TT genotype (P<0.05) (Fig. 1B).

## Discussion

The present study investigated the associations of endometrial cancer risk with the *MDM2* SNP309, *TP53* Arg72Pro, *ESR1 Pvu*II or *Xba*I and *p21* codon 31 polymorphisms in Japanese women. Although each polymorphism individually was unrelated to endometrial cancer risk, the SNP309 G allele was associated with an increased risk in women homozygous for the *TP53* codon 72 Arg allele. Furthermore, the relationship remained significant in a subgroup analysis (postmenopausal, type I endometrial cancer and overweight status. These were related to unopposed estrogen).

Few studies have addressed the association of SNP309 with the risk of endometrial cancer. Walsh *et al*(24) reported an OR of 2.76 for the GG vs. the TT genotype (95% CI, 1.06–7.20) in a United States case-control study. In a nested case-control study of Caucasian women, an OR of 1.87 (95% CI, 1.29–2.73) was reported for the GG genotype compared with the TT genotype (Nurses' Health Study, 454 cases and 1,132 controls; Women's Health Study, 137 cases and 411 controls) (25). Ashton *et al*(33), however, failed to document a similar association in an Australian study.

The association between the *TP53* Arg72Pro polymorphism and endometrial cancer risk has been investigated in several studies in Caucasians with inconsistent findings. The following findings have been reported for women of Asian descent: Ueda *et al*(27) reported an increased risk of endometrial cancer in Japanese patients harboring the Arg/Arg genotype compared to those with Arg/Pro and Pro/Pro genotypes combined. Niwa *et al*(34), however, found no such association. By contrast, having the Pro allele conferred an increased risk in Korean women (22).

Two studies have reported a potential interaction between the SNP309 G allele and the *TP53* Arg72Pro polymorphisms for endometrial cancer. Nunobiki *et al*(35) showed that the risk with the SNP309 GG vs. TT or TG was greater in women with Arg/Arg (OR, 3.28; 95% CI, 1.13–9.53) than in women with Arg/Pro or Pro/Pro of the *TP53* Arg72Pro (OR, 1.48; 95% CI, 0.62–3.52), but the interaction between the two SNPs was not evaluated.

Ashton *et al*(33) showed no effect of the *TP53* polymorphism and *MDM2* SNP309 alone or in combination on endometrial cancer risk. They did, however, show that the combination of SNP309 and *TP53* was associated with high-grade endometrial cancer (G2+G3). These observations are incompatible with the findings of the present study showing that the combination of SNP309 and *TP53* was associated with type I endometrial cancer (G1+G2). This discrepancy may have arisen due to the fact that G2 and G3 are classified as high-grade tumors. G3 tumors are a distinct entity. For example, in Japanese women, p53 mutations are significantly more common in G3 (43%) than in G1 (11%) tumors (36). p53 mutations are also detected more frequently in type II endometrial cancer. Tumors harboring p53 mutations are often more chemoresistant and have lower apoptotic rates. The status of the *TP53* gene (wild-type or mutant type) is critical when determining the relationship between grade and a *TP53* polymorphism. In the present study, an analysis of 102 cases that were not type II demonstrated a significant interaction between the *MDM2* SNP309 and *TP53* Arg72Pro polymorphism with endometrial cancer risk. We did not, however, ascertain whether the combination of *MDM2* and *TP53* Arg72Pro was associated with high-grade or non-endometrioid tumors as the overall number of these cases (G3, 10 cases; non-endometrioid, 13 cases) was small.

A major limitation of the present study was its small size. As most of the women with endometrial cancer are early stage and have positive outcomes, it was difficult to analyze the association between studied polymorphisms and disease outcome in the present study.

Five meta-analyses have been published addressing the *MDM2* SNP309 polymorphism and the risk of various types of tumor. Wilkening *et al*(37) combined the available genotype data for breast, colorectal and lung cancer. These results suggest that the SNP309 variant does not have an impact on the risk of breast or colorectal cancer. The OR for lung cancer, however, revealed an increased risk for GG vs. TT (OR, 1.27; 95% CI, 1.12–1.44). Hu *et al*(38) conducted a meta-analysis with different tumor types, including endometrial cancer. The OR revealed an increased risk for GG vs. TT (OR, 1.17; 95% CI, 1.04–1.33), and (OR, 1.37; 95% CI, 1.23–1.53) for the Asian population. Wan *et al*(39) performed a risk estimate with various tumor types. The OR for uterine cancer (eight studies) revealed an increased risk for GG vs. TT (OR, 1.34; 95% CI, 1.07–1.69) and (OR, 1.36; 95%CI, 1.18–1.56) for the Asian population (32 studies). This study included six studies (two for breast cancer, and one each for acute myeloid leukemia, hepatocellular carcinoma, lung cancer and gastric cancer) that explored interaction effects between *TP53* Arg72Pro and *MDM2* SNP309. They found that the combination of GG and Pro/Pro, TG and Pro/Pro and GG and Arg/Arg in comparison to the reference *MDM2* SNP309 TT and *TP53*Arg/Arg genotype, significantly increased the risk of cancer (OR, 3.38; 95% CI, 1.77–6.47), (OR, 1.88; 95% CI, 1.26–2.81) and (OR, 1.96; 95% CI, 1.01–3.78), respectively. Wo *et al*(40) completed a meta-analysis with multiple types of tumors, including four endometrial cancer studies. The OR reflected an increased risk of cancer for GG vs. TT (OR, 1.123; 95% CI, 1.056–1.193). Li *et al*(41) conducted a meta-analysis of 1,001 cases and 1,889 controls from six published case-control studies (24–26,33,35) to estimate the effect of SNP309 on endometrial cancer risk for GG vs. TT (OR, 1.54; 95% CI, 1.21–1.95). These analyses indicate that *MDM2* SNP309 serves as a tumor susceptibility marker, and that there is an association between *MDM2* SNP309 and *TP53* Arg/Pro regarding tumor susceptibility.

The other notable finding of the present study was that the combination of the *TP53* 72Pro allele and homozygosity for the *p21* codon 31 Ser allele was associated with a decreased risk of endometrial cancer. This is in contrast to a previous study showing that the combination of the *TP53* Pro allele and p21 Ser/Ser genotype significantly increased endometrial cancer risk (OR, 9.55; 95% CI, 4.40–21.24) (22). The result requires further investigation.

Finally, we investigated whether the SNP309 subtype modified the effect of RITA, an agent that blocks the p53-MDM2 interaction. RITA suppresses the proliferation of endometrial cancer cell lines. The effect was less pronounced in HHUA and Sawano cells containing the SNP309 G allele compared with that in Hec6 cells, which harbor the TT genotype. Arva *et al*(42) showed that cells carrying the SNP309 GG genotype exhibited a compromised *TP53* response pathway and formed transcriptionally inactive p53-MDM2 complexes in response to stress. These results support our findings. Additional functional analyses are required to fully elucidate the effects of these polymorphisms on the effect of agents such as RITA on endometrial cancer cell lines.

In conclusion, endometrial cancer is characterized by numerous genetic alterations, including those in p53, K-ras, *PTEN* and β-catenin. We previously demonstrated that the Ras/ER/MDM2 pathway is important for proliferation of endometrial cancer cells *in vitro*. We demonstrated in this case-control study that polymorphisms of *TP53* and *MDM2* modify the effect of this signaling pathway and thus increase the risk of endometrial cancer.

The increased incidence of endometrial cancer in Japan may reflect changes in lifestyle. The interplay between genetic and environmental factors is now being investigated in the context of gynecologic cancer. It is well known that endometrial cancer risk includes an environmental component. Further studies are necessary to elucidate the genetic components of risk. Understanding this relationship is the first step towards developing new methods of endometrial cancer prevention and treatment.

## Figures and Tables

**Table I:** Characteristics of cases and controls.

Characteristics	Cases, n=125	Controls, n=200	P-value
Age (years, median)	27–88 (56)	49–75 (59)	<0.0001
Menopausal status (%)			
Premenopausal	47 (37.6)	17 (8.5)	<0.05
Postmenopausal	78 (62.4)	183 (91.5)	<0.05
Parity (%)			
Never	37 (29.6)	..20 (10.0)	<0.05
Ever	88 (70.4)	180 (90.0)	<0.05
BMI (kg/m^2^) mean (SD)	24.2 (5.0)	22.9 (3.0)	<0.0001
<25 (%)	76 (60.8)	162 (81.0)	
≥25 (%)	49 (39.2)	..38 (19.0)	
FIGO stage (%)			
I	95 (76.0)		
II	7 (5.6)		
III	19 (15.2)		
IV	3 (2.4)		
Unknown^a^	1 (0.8)		
Histology			
Endometrioid adenocarcinoma (%)	112 (89.6)		
Grade 1	74		
Grade 2	28		
Grade 3	10		
Non-endometrioid adenocarcinoma (%)	13 (10.4)		
Serous papillary adenocarcinoma	4		
Clear cell adenocarcinoma	3		
Mixed^b^	3		
Undifferentiated	1		
Neuroendocrine carcinoma	1		
Squamous cell carcinoma	1		

SD, standard deviation; BMI, body mass index;

a no surgery.

b Endometrioid adenocarcinoma + clear cell adenocarcinoma: two. Endometrioid adenocarcinoma + serous papillary adenocarcinoma: one.

**Table II:** *MDM2*, *TP53*, *ESR1* and *p21* genotypes and endometrial cancer risk.

Genotype	Cases, n=125 (%)	Controls, n=200 (%)	Crude OR (95% CI)	Adjusted^a^ OR (95% CI)
*MDM2* SNP309				
TT	30 (24.0)	62 (31.0)	1.00 (reference)	1.00 (reference)
TG	61 (48.8)	98 (49.0)	1.29 (0.75–2.21)	1.09 (0.60–1.99)
GG	34 (27.2)	40 (20.0)	1.76 (0.93–3.30)	1.64 (0.81–3.28)
			P-trend=0.08	P-trend=0.45
TG+GG	95 (76.0)	138 (69.0)	1.42 (0.86–2.37)	1.24 (0.70–2.18)
*TP53* codon 72				
Arg/Arg	52 (41.6)	75 (37.5)	1.00 (reference)	1.00 (reference)
Arg/Pro	55 (44.0)	102 (51.0)	0.78 (0.48–1.26)	0.66 (0.38–1.15)
Pro/Pro	18 (14.4)	23 (11.5)	1.13 (0.55–2.30)	1.17 (0.54–2.55)
			P-trend=0.08	P-trend=0.79
Arg/Pro + Pro/Pro	73 (58.4)	125 (62.5)	0.84 (0.53–1.33)	0.76 (0.46–1.27)
*ESR1 Pvu*II				
TT	38 (30.4)	60 (30.0)	1.00 (reference)	1.00 (reference)
TC	60 (48.0)	102 (51.0)	0.93 (0.55–1.56)	0.94 (0.55–1.56)
CC	27 (21.5)	38 (19.0)	1.12 (0.59–2.13)	0.89 (0.43–1.86)
			P-trend=0.78	P-trend=0.76
TC+CC	87 (69.6)	140 (70.0)	0.98 (0.60–1.60)	0.93 (0.53–1.60)
*ESR1 Xba*I				
AA	77 (61.6)	127 (63.5)	1.00 (reference)	1.00 (reference)
AG	42 (33.6)	64 (32.0)	1.08 (0.67–1.75)	0.88 (0.51–1.53)
GG	6 (4.8)	9 (4.5)	1.10 (0.38–3.21)	0.60 (0.16–2.24)
			P-trend=0.74	P-trend=0.43
AG+GG	48 (38.4)	73 (36.5)	1.09 (0.68–1.72)	0.84 (0.50–1.43)
*p2*1 codon 31				
Ser/Ser	21 (16.8)	38 (19.0)	1.00 (reference)	1.00 (reference)
Ser/Arg	73 (58.4)	109 (54.5)	1.21 (0.66–2.23)	1.13 (0.57–2.22)
Arg/Arg	31 (24.8)	53 (26.5)	1.06 (0.53–2.12)	1.01 (0.47–2.19)
			P-trend=0.95	P-trend=0.98
Ser/Arg + Arg/Arg	104 (83.2)	162 (81.0)	1.16 (0.65–2.09)	1.09 (0.57–2.09)

OR, odds ratio;

a adjusted for age and BMI.

**Table III:** *MDM2* and *TP53* genotypes and endometrial cancer risk.

Genotype	No. of cases (%)	No. of controls (%)	Crude OR (95% CI)	Adjusted^a^ OR (95% CI)
Postmenopause	n=78	n=183		
*MDM2* SNP309				
TT	22 (28.2)	57 (31.1)	1.00 (reference)	1.00 (reference)
TG	36 (46.2)	90 (49.2)	1.04 (0.55–1.94)	1.12 (0.59–2.15)
GG	20 (25.6)	36 (19.7)	1.44 (0.69–3.00)	1.60 (0.75–3.43)
			P-trend=0.36	P-trend=0.24
TG + GG	56 (71.8)	126 (68.9)	1.15 (0.64–2.07)	1.26 (0.69–2.31)
*TP53* codon 72				
Arg/Arg	33 (42.3)	69 (37.7)	1.00 (reference)	1.00 (reference)
Arg/Pro	32 (41.0)	93 (50.8)	0.72 (0.40–1.28)	0.79 (0.43–1.43)
Pro/Pro	13 (16.7)	21 (11.5)	1.29 (0.58–2.90)	1.44 (0.63–3.31)
			P-trend= 0.95	P-trend= 0.70
Arg/Pro + Pro/Pro	45 (57.7)	114 (62.3)	0.83 (0.48–1.42)	0.91 (0.52–1.58)
Type1^b^				
*MDM2* SNP309	n=102	n=200		
TT	26 (25.5)	62 (31.0)	1.00 (reference)	1.00 (reference)
TG	47 (46.1)	98 (49.0)	1.14 (0.64–2.03)	0.88 (0.46–1.72)
GG	29 (28.4)	40 (20.0)	1.73 (0.89–3.35)	1.56 (0.73–3.33)
			P-trend=0.11	P-trend=0.30
TG + GG	76 (74.5)	138 (69.0)	1.31 (0.76–2.25)	1.07 (0.58–1.97)
*TP53* codon 72				
Arg/Arg	45 (44.1)	75 (37.5)	1.00 (reference)	1.00 (reference)
Arg/Pro	44 (43.1)	102 (51.0)	0.79 (0.43–1.20)	0.55 (0.30–1.02)
Pro/Pro	13 (12.8)	23 (11.5)	0.94 (0.43–2.04)	1.00 (0.42–2.38)
			P-trend=0.51	P-trend=0.43
Arg/Pro + Pro/Pro	57 (55.9)	125 (62.5)	0.76 (0.47–1.23)	0.64 (0.36–1.12)
Postmenopause + type 1	n=59	n=183		
*MDM2* SNP309				
TT	19 (32.2)	57 (31.1)	1.00 (reference)	1.00 (reference)
TG	25 (42.4)	90 (49.2)	0.83 (0.42–1.65)	0.94 (0.46–1.93)
GG	15 (25.4)	36 (19.7)	1.25 (0.56–2.77)	1.50 (0.64–3.47)
			P-trend= 0.66	P-trend=0.40
TG + GG	40 (67.8)	126 (69.9)	0.95 (0.51–1.79)	1.09 (0.56–2.13)
*TP53* codon 72				
Arg Arg	28 (47.5)	69 (37.7)	1.00 (reference)	1.00 (reference)
Arg/Pro	22 (37.3)	93 (50.8)	0.58 (0.31–1.11)	0.66 (0.34–1.30)
Pro/Pro	9 (15.2)	21 (11.5)	1.06 (0.43–2.59)	1.24 (0.49–3.16)
			P-trend=0.55	P-trend=0.89
Arg/Pro + Pro/Pro	31 (52.5)	114 (62.3)	0.67 (0.37–1.21)	0.77 (0.41–1.43)

OR, odds ratio;

a adjusted for age and BMI.

b Type 1; endometrioid adenocarcinoma G1 and G2.

**Table IV:** Endometrial cancer risk for combined effect of *MDM2* and *TP53* genotypes.

Genotype		No. of cases (%)	No. of controls (%)	Crude OR (95% CI)	Adjusted^a^ OR (95% CI)
All		n=125	n=200		
*MDM2* SNP309	*TP53* codon 72				
TT	Arg/Arg	10 (8.0)	28 (14.0)	1.00 (reference)	1.00 (reference)
TT	Arg/Pro + Pro Pro	20 (16.0)	34 (17.0)	1.65 (0.66–4.09)	1.79 (0.68–4.73)
TG + GG	Arg/Arg	42 (33.6)	47 (23.5)	2.50 (1.09–5.75)	2.53 (1.03–6.21)
TG + GG	Arg/Pro + Pro/Pro	53 (42.4)	91 (45.5)	1.63 (0.73–3.62)	1.35 (0.56–3.24)
				P-interaction=0.09	P-interaction=0.04
Postmenopause		n=78	n=183		
*MDM2* SNP309	*TP53* codon 72				
TT	Arg/Arg	6 (7.7)	25 (13.7)	1.00 (reference)	1.00 (reference)
TT	Arg/Pro + Pro/Pro	16 (20.5)	32 (17.5)	2.08 (0.71–6.10)	2.46 (0.81–7.44)
TG + GG	Arg/Arg	27 (34.6)	44 (24.0)	2.56 (0.93–7.03)	2.96 (1.04–8.44)
TG + GG	Arg/Pro + Pro/Pro	29 (37.2)	82 (44.8)	1.47 (0.55–3.95)	1.84 (0.66–5.16)
				P-interaction=0.04	P-interaction=0.03
Type 1^b^		n=102	n=200		
*MDM2* SNP309	*TP53* codon 72				
TT	Arg/Arg	9 (8.8)	28 (14.0)	1.00 (reference)	1.00 (reference)
TT	Arg/Pro + Pro/Pro	17 (16.7)	34 (17.0)	1.55 (0.60–4.02)	1.82 (0.65–5.11)
TG + GG	Arg/Arg	36 (35.3)	47 (23.5)	2.38 (1.00–5.67)	2.51 (0.97–6.53)
TG + GG	Arg/Pro + Pro/Pro	40 (39.2)	91 (45.5)	1.37 (0.59–3.16)	0.98 (0.37–2.58)
				P-interaction=0.07	P interaction=0.01
Postmenopause + Type 1		n=59	n=183		
*MDM2* SNP309	*TP53* codon 72				
TT	Arg/Arg	5 (8.5)	25 (13.7)	1.00 (reference)	1.00 (reference)
TT	Arg/Pro + Pro/Pro	14 (23.7)	32 (17.5)	2.19 (0.70–6.89)	2.76 (0.83–9.19)
TG + GG	Arg/Arg	23 (39.0)	44 (24.0)	2.61 (0.88–7.73)	3.24 (1.03–10.16)
TG + GG	Arg/Pro + Pro/Pro	17 (28.8)	82 (44.8)	1.04 (0.35–3.09)	1.43 (0.45–4.57)
				P-interaction=0.01	P interaction=0.009

OR; odds ratio; CI; confidence interval.

a Adjusted for age and BMI.

b Type 1, endometrioid adenocarcinoma G1 and G2.

**Table V:** Endometrial cancer risk for combined effect of *MDM2*, *TP53*, *ESR1* and *p21* genotypes.

Genotype		Cases, n=125 (%)	Controls, n=200 (%)	Crude OR (95% CI)	Adjusted^a^ OR (95% CI)
*MDM2* SNP309	*ESR1 Pvu*II				
TT	TT	8 (6.4)	20 (10.0)	1.00 (reference)	1.00 (reference)
TT	TC + CC	22 (17.6)	42 (21.0)	1.31 (0.50–3.45)	0.96 (0.34–2.69)
TG + GG	TT	30 (24.0)	40 (20.0)	1.86 (0.73–4.83)	1.29 (0.47–3.57)
TG + GG	TC + CC	65 (52.0)	98 (49.0)	1.66 (0.69–3.99)	1.18 (0.46–2.99)
				P-interaction=0.49	P-interaction=0.93
*MDM2* SNP309	*ESR1 Xba*I				
TT	AA	20 (16.0)	42 (21.0)	1.00 (reference)	1.00 (reference)
TT	AG + GG	10 (8.0)	20 (10.0)	1.05 (0.42–2.65)	0.83 (0.30–2.23)
TG + GG	AA	57 (45.6)	85 (42.5)	1.41 (0.75–2.64)	1.25 (0.63–2.47)
TG + GG	AG + GG	38 (30.4)	53 (26.5)	1.51 (0.77–2.96)	1.05 (0.49–2.23)
				P interaction=0.97	P-interaction=0.96
*MDM2* SNP309	*p21* codon 31				
TT	Ser/Ser	8 (6.4)	11 (5.5)	1.00 (reference)	1.00 (reference)
TT	Ser/Arg + Arg/Arg	22 (17.6)	51 (25.5)	0.59 (0.21–1.68)	0.58 (0.19–1.75)
TG + GG	Ser/Ser	13 (10.4)	27 (13.5)	0.66 (0.22–2.04)	0.58 (0.17–2.00)
TG + GG	Ser/Arg + Arg/Arg	82 (65.6)	111 (55.5)	1.02 (0.39–2.64)	0.87 (0.31–2.41)
				P-interaction=0.14	P-interaction=0.18
*TP53* codon 72	*p21* codon 31				
Arg/Arg	Ser/Ser	16 (12.8)	18 (9.0)	1.00 (reference)	1.00 (reference)
Arg/Arg	Ser/Arg + Arg/Arg	36 (28.8)	57 (28.5)	0.71 (0.32–1.57)	0.85 (0.35–2.02)
Arg/Pro + Pro/Pro	Ser/Ser	5 (4.0)	20 (10.0)	0.28 (0.09–0.92)	0.39 (0.11–1.43)
Arg/Pro + Pro/Pro	Ser/Arg + Arg/Arg	68 (54.4)	105 (52.5)	0.73 (0.35–1.53)	0.72 (0.32–1.64)
				P-interaction=0.04	P-interaction=0.27

OR, odds ratio; CI, confidence interval.

a Adjusted for age and BMI.

**Table VI:** Genotype of endometrial cancer cell lines.

Cell line	*MDM2* SNP309	*TP53* codon 72	*TP53* status
Hec6	TT	Arg/Arg	Wild-type
HHUA	TG	Arg/Arg	Wild-type
Sawano	GG	Arg/Pro	Wild-type
